# iTRAQ-Based Proteomics to Reveal the Mechanism of Hypothalamus in Kidney-Yin Deficiency Rats Induced by Levothyroxine

**DOI:** 10.1155/2019/3703596

**Published:** 2019-03-04

**Authors:** Wei Guan, Yan Liu, Xiaomao Li, Bingyou Yang, Haixue Kuang

**Affiliations:** Key Laboratory of Chinese Materia Medica (Ministry of Education), Heilongjiang University of Chinese Medicine, Harbin 150040, China

## Abstract

Kidney-yin deficiency syndrome (KYDS) is a typical syndrome encountered in traditional Chinese medicine (TCM) and is characterized by impaired lipid and glucose homeostasis. The hypothalamus acts as an important regulatory organ by controlling lipid and glucose metabolism in the body. Therefore, proteins in the hypothalamus could play important roles in KYDS development; however, the mechanisms responsible for KYDS remain unclear. Herein, iTRAQ-based proteomics was performed to analyze the protein expression in the hypothalamus of KYDS rats induced by levothyroxine (L-T_4_). Results revealed a total of 44 downregulated and 18 upregulated proteins in KYDS group relative to the control group. Gene Ontology (GO) analysis revealed that the differently expressed proteins (DEPs) were related to single-organism metabolism process under the biological process (BP), extracellular region part and organelle under the cellular component (CC), and oxidoreductase activity under the molecular function (MF). Kyoto Encyclopedia of Gene and Genomes (KEGG) analysis showed that fatty acid degradation and pyruvate metabolism participated in the metabolism regulation in KYDS rats. RT-PCR validation of five distinctly expressed proteins related to the two pathways was consistent with the results of proteomics analysis. Taken together, the inhibition of fatty acid degradation and pyruvate metabolism in hypothalamus could potentially cause the dysfunction of the lipid and glucose metabolism in KYDS rats. This current study identified some novel potential biomarkers of KYDS and provided the basis for further research of KYDS.

## 1. Introduction

KYDS is one of the typical syndromes in TCM that is caused by kidney-yin insufficiency, and flaming of asthenia-fire. KYDS is characterized by dizziness, tinnitus, flaccid waist and knees, hectic fever, dry mouth and throat, night sweat, spermatorrhea, thirst, thread, and rapid pulse [1]. The increasing pace of life makes people physically and mentally exhausted with subhealth status, such as dysphoria, neurasthenia, hyperphagia, insomnia, and alopecia, which belong to KYDS [2]. The dysregulation of lipid and glucose metabolism consistently observed in KYDS patients and can lead to hyperthyroidism, diabetes, immunological dysfunction, and other diseases commonly encountered in modern medical research [3, 4]. KYDS always induces mitochondrial defects, which in turn leads to the impairment of the tricarboxylic acid cycle and ultimately results in energy metabolic disorders [5]. Likewise, previous studies indicated that KYDS alters the organic acids generated by intestinal microflora, which are important for the maintenance of lipid and glucose homeostasis in the body and influence the energy metabolism [6].

The hypothalamic-pituitary-thyroid axis plays a key role in the neuroendocrine system, which is closely associated with the KYDS pathogenesis [7, 8]. The hypothalamus is located below the thalamus and contains small nucleuses, which is known to be involved in various functions. The hypothalamus links the nervous system to the endocrine system via hypophysis and synthesis or secretion of releasing hormones or hypothalamic hormones that regulate automatic nervous system activities and metabolic processes, especially lipid and glucose metabolism [9–13]. Given that KYDS is closely associated with the disruption of lipid and glucose metabolism, we hypothesized that KYDS alters the protein expression with regulatory functions in the hypothalamus, which ultimately disrupt lipid and glucose homeostasis. Although KYDS has been investigated in clinical or experimental research for decades, the mechanisms underlying disease development and progression remain unclear.

The unique advantages of TCM in treating diseases have been demonstrated for thousands of years. However, there are some limitations that hinder research on TCM syndromes because of the abstract theories and complex pathogenesis in TCM. Along with the development of modern techniques, the emergence of systems biology opens up new avenues for investigating TCM syndromes [14]. Proteomics, similar to TCM in theory, is a major branch of systems biology that deals with the analysis of large-scale protein expression profiles in cells or tissues. Proteomics can aid in the discovering of biomarkers and the mechanisms of underlying diseases [15]. Many techniques have been applied in proteomics, including isobaric tags for relative and absolute quantitation (iTRAQ), an isobaric labelling method employed in quantitative proteomics by tandem mass spectrometry. iTRAQ has been widely employed in TCM research because of advantages, such as higher sensitivity, specificity, and selectivity [8, 16–18]. Advances in proteomics can improve TCM research [19].

In the present study, iTRAQ combined with two-dimensional liquid chromatography tandem mass spectra analysis was employed to determine the protein profiles of the hypothalamus in the L-T_4_ induced KYDS rat model. Our findings provided deeper insights into the pathological essence of KYDS and provide a strong basis for further research on KYDS.

## 2. Materials and Methods

### 2.1. Chemicals and Reagents

iTRAQ® Reagents Multiplex Kit and Ammonium formate were purchased from Sigma (CA, USA). Levothyroxine sodium tablets (L-T_4_) were obtained from MerckSerono (Darmstadt, Germany). Saline solution was purchased from Medlian Pharmaceutical Co., Ltd. (Harbin, China). Tissue Protein Extraction Kit and BCA Protein Assay Kit were obtained from CWbiotech Co., Ltd. (Shanghai, China). Triiodothyronine (T_3_) Assay Kit and Thyroxine (T_4_) Assay Kit were purchased from Nanjing Jiancheng Bioengineering Institute (Nanjing, China). The estrogen (E_2_), testosterone (T), cAMP, and cGMP ELISA kits were obtained from USCN Life Science Inc. (Wuhan, China). Trypsin was purchased from Promega Corporation (Wisconsin, USA). Vivacon 500 tubes were purchased from Satorius (Gottingen, Germany). TRIzol reagent was obtained from Life technologies (New York, USA). The reverse transcription kit and fluorescent dye were obtained from Roche (Switzerland). All primers were purchased from ComateBio Company (Jilin, China). Chromatographic grade acetonitrile, methanol, and ultrapure water were obtained from Thermo Fisher (MA, USA).

### 2.2. Animals and Treatments

Male Sprague Dawley (SD) rats [SCXK (Heilongjiang) 2016009] weighing 180-220 g were purchased from the Animal Center Building of Heilongjiang University of TCM. All animals were approved by the Institutional Ethics Committee of Heilongjiang University of Chinese Medicine and all experimental procedures were performed in accordance with the Declaration of Helsinki. All rats were provided with clean drinking water, kept in air-conditioned room (25 ± 2°C), and acclimatized for 7 days with 12 h light/dark cycle. For the preparation of L-T_4_, the levothyroxine sodium tablets were dissolved in distilled water to a concentration of 1.2 mg/mL. After acclimation for 7 days, 24 male SD rats were randomly divided into the following two groups, 12 rats for the control group and 12 rats for the KYDS group. The rats in the KYDS group were intragastrically administered with the L-T_4_ suspension (12.0 mg/kg), whereas rats in the control group were gavaged with the same amount of saline every morning for 21 days. After 21 days, all rats were anesthetized by intraperitoneal injection with 10% chloral hydrate in a dose of 350 mg/kg. The blood samples were collected from the abdominal aorta and clotted at the room temperature (25 ± 2°C). The serum was separated by centrifugation (3500 rpm for 15 min at 4°C) and stored at -20°C for biochemical analysis. Excised tissue samples from the hypothalamus (100 mg) were directly flash frozen in liquid nitrogen and then stored at -80°C for further analysis.

### 2.3. Biochemical Analysis and KYDS Status

Serum samples from each treatment group were thawed at the room temperature (25 ± 2°C). The serum concentrations of T_3_, T_4_, E_2_, T cAMP, and cGMP were measured on a microplate reader using the ELISA assay kits following the manufacturer's instructions (PerkinElmer, Waltham, USA). The body weights and the temperatures of the rats were measured.

### 2.4. Protein Isolation and Labelling with the iTRAQ Reagent

To establish two biological replicates, 12 samples in each group were randomly divided into two subgroups. All samples were mixed together in each subgroup; therefore, four pooling samples were assembled. The pooled samples were ground into powder in liquid nitrogen. The proteins in the powder were extracted by using a tissue protein extraction kit. The concentrations of the extracted proteins were measured on a microplate reader using BCA protein assay kit.

The total proteins (100 *μ*g) from each subgroup were digested in trypsin. After trypsin digestion, the peptides were labelled with iTRAQ reagent-8-plex multiplex kit following the manufacturer's instructions after trypsin digestion. For duplication the subgroups of the control group were labelled with 113 and 119 tags, while the subgroups from the model group were labelled with 114 and 121 tags. All labelled samples were mixed and dried by vacuum centrifugation (EYELA, Tokyo, Japan).

### 2.5. High-pH Reverse-Phase Liquid Chromatography Fraction

The peptides labelled with the iTRAQ reagent were separated on an HPLC system (Waters, USA) with the combination of Waters XBridge C18 (4.6 mm×250 mm, 5 *μ*m). The dried peptides were then redissolved in buffer A (20 mM ammonium formate, pH 10) and vortexed at 12,000×g for 20 min. The supernatant was loaded onto the HPLC column. The peptides were eluted at a 1 mL/min with a gradient of 5% buffer B (20 mM ammonium formate in 80% acetonitrile, pH 10) for 5 min, 5%-15% buffer B for 25 min, 15%-38% buffer B for 15 min, 38%-90% buffer B for 1 min, and 90% buffer B continuously eluted for a period of 8.5 min, 90%-5% buffer B for 0.5 min, and 5% buffer B for 10 min. The eluted fractions were collected after 5 min at 1-min intervals and divided into ten fractions. All ten fractions were dried by vacuum centrifugation.

### 2.6. LC-MS/MS Analysis

LC-MS/MS analysis was carried out on an AB SCIEX nanoLC-MS/MS (Triple TOF 5600) system (AB SCIEX, USA). All the ten fractions were dissolved in 30 *μ*L of mobile phase A (0.1% formic acid, 2% acetonitrile) and centrifuged at 12,000×g for 20 min. Afterwards, 20 *μ*L of each samples were separated in an analytical column (C18-CL-120, 0.075 mm×150 mm, 3 *μ*m) with an 80 min solvent gradient from 5-80% buffer B (0.1% formic acid, 98% acetonitrile) at a flow rate of 0.3 *μ*L/min. MS1 spectra were collected in the range 350-1250 m/z for 250 ms. The 30 precursors with the strongest signals were selected for fragmentation. MS2 spectra were collected in the range of 100-1500 m/z for 100 ms.

### 2.7. MS Data Processing and Analysis

Raw MS data were imported into the AB ProteinPilot™ v4.5 software (AB SCIEX, USA) for protein identification using the Paragon algorithm. The proteins with at least one unique peptide and more than 1.3 unused values were selected for further identification, and proteins with at least two peptides were considered for further analysis.* P* ≤ 0.05 and fold-changes lower than 0.67 or higher than 1.5 were considered as significant.

### 2.8. Bioinformatics Analysis

Functional classification and annotation were performed with Gene Ontology (GO) using the DAVID bioinformatics resources 6.8 (http://david.ncifcrf.gov/) to determine enrichment in cellular components (CC), biological process (BP) and molecular function (MF). Pathway analysis was performed using the Kyoto Encyclopedia of Gene and Genomes (KEGG) (http://www.genome.ad.jp/kegg/mapper.html) pathway to identify significant enrichment of biochemical pathways and molecular interactions. Functional protein association networks were generated using STRING.

### 2.9. RT-PCR Analysis

The total RNA from each hypothalamic tissue sample was extracted using the TRIzol reagent. The ratio of the total RNA at 260 nm to the absorption at 280 nm was measured using a NanoDrop ND-8000 (Thermo, Waltham, MA, USA) instrument to determine the RNA purity. The total RNA with higher purity was transcribed into cDNA on a S1000TM Thermal Cycler (Bio-Rad, Hercules, USA) instrument using a reverse transcription kit following the manufacturer's instructions. Then, the total RNA extracted from the hypothalamic tissue was quantified in real time using a fluorescent dye on a real-time thermal cycler (Bio-Rad, Hercules, USA). All primer sequences were designed by Sangon Biotech (Shanghai, China) (https://www.sangon.com) and are shown in Table 1. The fold-change values were normalized using GAPDH levels.

### 2.10. Statistical Analysis

Statistical analysis was performed using GraphPad Prism software (GraphPad software, San Diego, CA). Data were presented as mean ± SD. Student's* t*-test was performed to determine statistically significant differences between the two groups.* P* < 0.05 was considered statistically significant.

## 3. Results

### 3.1. Confirmation of Kidney-Yin Deficiency Syndrome Status

**Figure 1:**
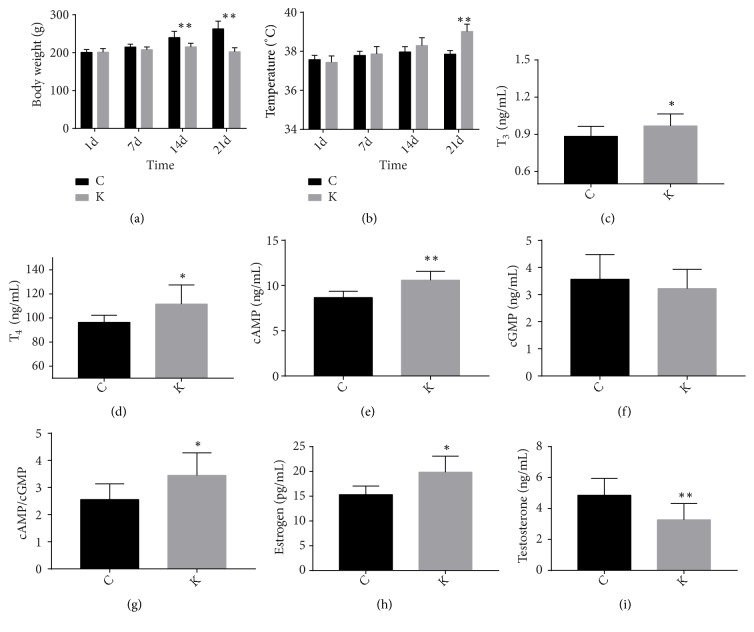
Comparison of body weight, temperature, serum T_3_, T_4_, cAMP, cGMP, cAMP/cGMP, E_2_, and T levels between the control (C) and KYDS (K) groups. Data were expressed as means ± SD, ^*∗*^*P *< 0.05, and ^*∗∗*^*P* < 0.01 versus the control group. (a) Body weight; (b) temperature; (c) serum T_3_; (d) serum T_4_; (e) serum cAMP; (f) serum cGMP; (g) cAMP/cGMP; (h) serum E2; (i) serum T.

Rats in the KYDS group showed irritable hyperhidrosis and tachypnea. The body weights of the KYDS group were significantly lower on the 14th day compared to those of rats in the control group (*P* < 0.01) (Figure 1(a)). The body temperatures of the rats in KYDS group started to increase from the 14th day and were markedly elevated on the 21st day comparing to those of rats in the control group (*P* < 0.01) (Figure 1(b)). Biochemical analysis revealed that serum T_3_ and T_4_ levels were significantly higher in the KYDS group than in the control group (*P *< 0.05) (Figures 1(c) and 1(d)). The cAMP levels and cAMP/cGMP ratio were markedly increased in KYDS rats (*P* < 0.01,* P* < 0.05) (Figures 1(e) and 1(g)); however we observed no differences in cGMP levels between the two groups (Figure 1(f)). The E_2_ concentrations in KYDS group were significantly higher than those in the control group (*P* < 0.05); on the other hand, the serum T levels in KYDS rats were markedly lower than those in rats in control group (*P* < 0.01) (Figures 1(h) and 1(i)).

### 3.2. iTRAQ Quantification of Differentially Expressed Proteins

A total of 1735 proteins were identified after merging data from two replicates using ProteinPilot software, among which 831 proteins contained at least 1 unique peptide with unused values less than 1.3. Compared to the control group, the proteins with fold-change > 1.5 or < 0.67 with more than two peptides were regarded as the most differently expressed proteins (*P* < 0.05). Results showed that 44 and 18 proteins were markedly downregulated and upregulated between the model and control groups, respectively.

### 3.3. Bioinformatics Analysis

**Figure 2:**
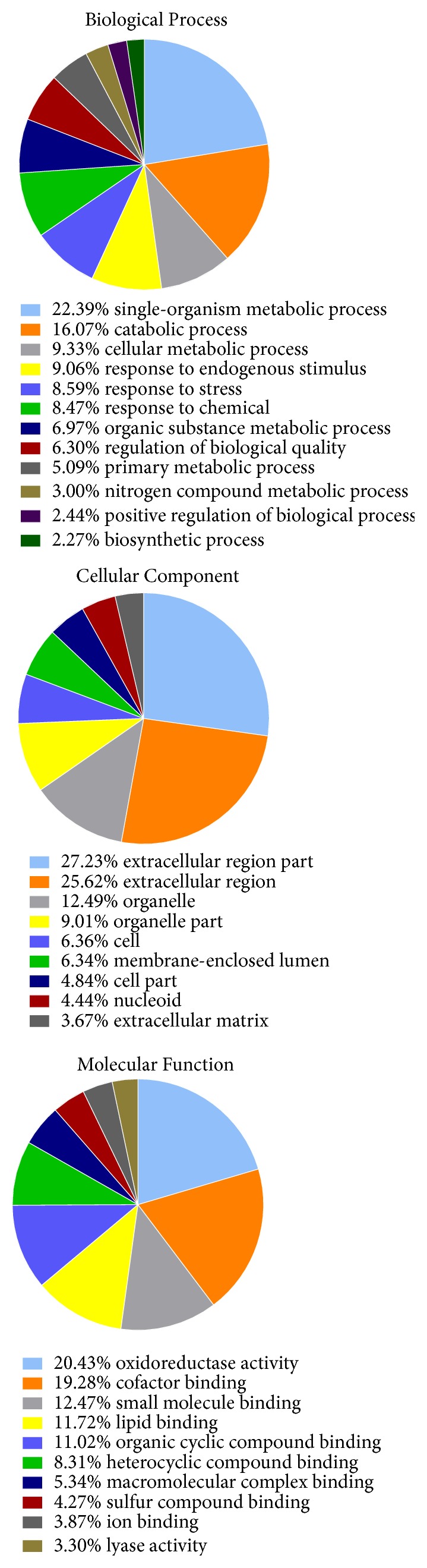
Go analysis for functional classification of the differently expressed proteins, (a) biological process; (b) cellular component; (c) molecular function.

GO analysis can help understand the functional classifications of all identified DEPs. In this study, GO analysis was performed using the DAVID bioinformatics resource 6.8 to categorize the proteins based on biological process (BP), molecular function (MF), and cellular component (CC) categories. BP analysis revealed that the majority of the proteins were primarily involved in the single-organism metabolic process (22%), catabolic process (16%), cellular metabolic process (9%), response to endogenous stimulus (9%), response to stress (9%), response to chemical (9%), and organic substance metabolic process (7%) (Figure 2(a)). CC analysis showed that the DEPs were primarily located in the extracellular region part (27%), extracellular region (26%), organelle (13%), organelle part (9%), cell (6%), membrane enclosed lumen (6%), cell part (5%), nucleoid (4%), and extracellular matrix (4%) (Figure 2(b)). According to MF analysis, DEPs were involved in the oxidoreductase activity (21%), cofactor binding (19%), small molecule binding (13%), lipid binding (12%), organic cyclic compound binding (11%), heterocyclic compound binding (8%), macromolecular complex binding (5%), sulfur compound binding (4%), ion binding (4%), and lyase activity (3%) (Figure 2(c)).

### 3.4. KEGG Pathways and STRING Analysis

**Figure 3:**
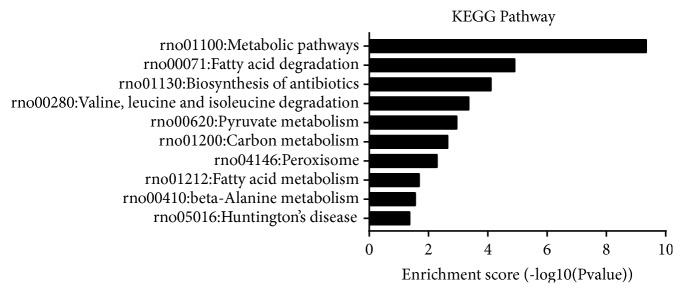
Top ten significantly enriched pathways identified by KEGG pathway analysis.

**Figure 4:**
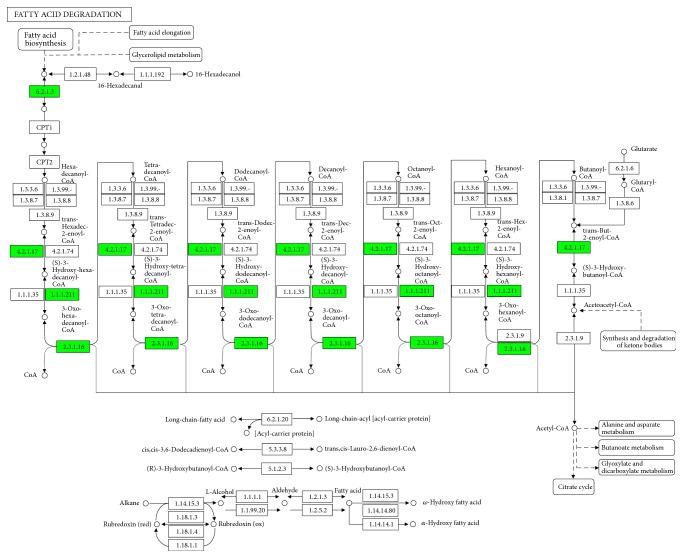
KEGG pathway analysis. Results show proteins involved in the fatty acid degradation pathway. Green colors represent the proteins that are downregulated in the KYDS group.

**Figure 5:**
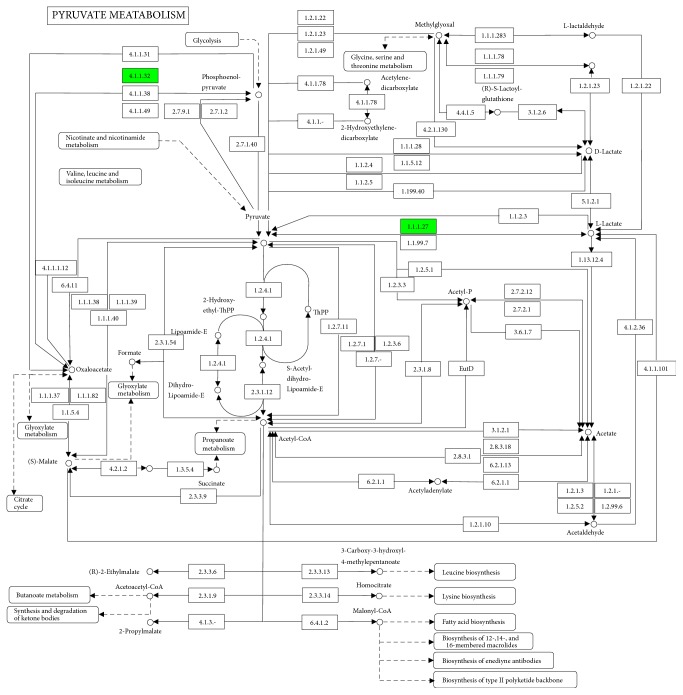
KEGG pathway analysis. Results show proteins involved in the pyruvate metabolism pathway. Green colors represent the proteins that are downregulated proteins in the KYDS group.

**Figure 6:**
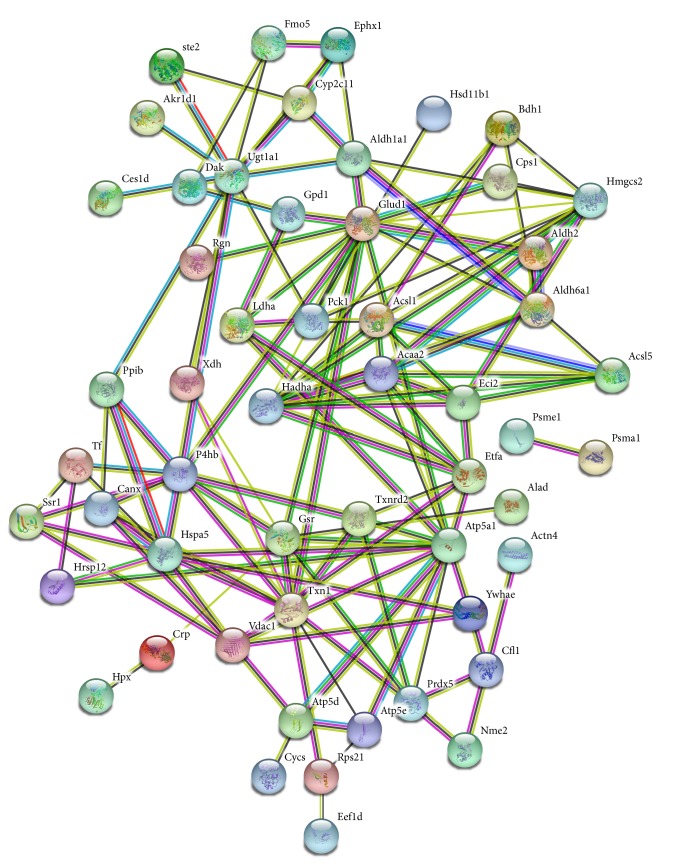
The directive protein-protein interaction analysis of differently expressed proteins using STRING.

KEGG pathway analysis is widely conducted in proteomics studies to identify the functions of proteins. KEGG pathway analysis revealed that 26 pathways were enriched in the KYDS group, with top ten pathways showing statistically significant enrichment (Figure 3). Of these, fatty acid metabolism (Figure 4) and pyruvate metabolism (Figure 5) were found to be associated with glucose and lipid homeostasis. STRING analysis could visually help us understand the interactions between these DEPs. Results of STRING analysis revealed that 3-ketoacyl-CoA thiolase (Acaa2), long-chain acyl-CoA synthetase 1 (Acsl1), trifunctional enzyme subunit alpha (Hadha), L-lactate dehydrogenase A chain (Ldha), and cytosolic phosphoenolpyruvate carboxykinase (Pck1) correspond to critical nodes in the network (Figure 6).

### 3.5. Gene Expression Analysis of the Differently Expressed Proteins

**Figure 7:**
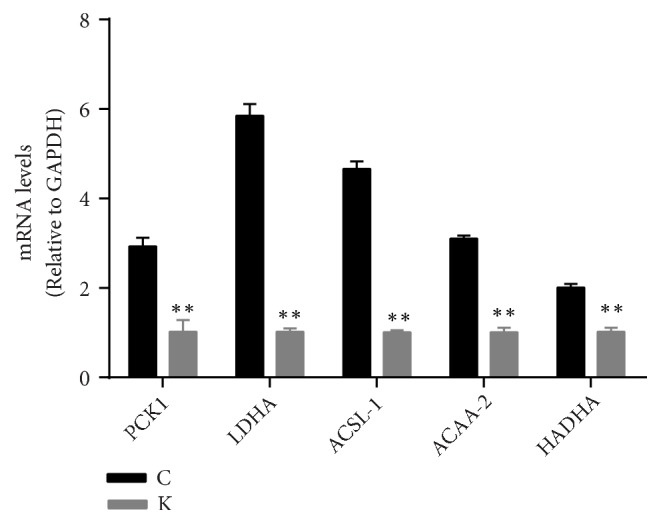
mRNA levels of Pck1, Ldha, Acsl1, Acaa2, and Hadha in KYDS (K) group compared to the control group (C). Data were expressed as means ± SD; ^*∗∗*^*P* < 0.01 compared with the control group.

To determine whether the protein expression levels of these DEPs in the KYDS group were consistent with the mRNA levels, RT-PCR was performed on five key proteins (Acaa2, Acsl1 Hadha, Ldha, and Pck1) that were primarily associated with fatty acid and glucose metabolism. Results showed that the expression patterns of these five genes were consistent with the protein levels determined based on proteomics analysis (Figure 7).

## 4. Discussion

KYDS is a common syndrome encountered in TCM, therefore elucidating the mechanisms and biological essences underlying KYDS can provide important insights for the treatment of the disease. Previous studies have demonstrated that administration with thyroxine, thyroxine with reserpine, febricity herbs can induce KYDS, among which thyroxine is commonly applied to simulate KYDS in the rat model [6, 20–23]. Extensive studies reported in literatures indicated that the rats subjected to gastric administration of levothyroxine for 21 days, consistently showed weight loss, high temperature, dysphoria, irritability, and other symptoms that were similar to the clinical manifestations of KYDS in TCM [1, 6, 21, 24]. Hyperactivity of the hypothalamus-pituitary-thyroid axis (HPT) is always observed during KYDS, in addition, T_3_ and T_4_ levels, which reflect the functional state of the HPT, are significantly dysregulated in KYDS [25–29]. The kidney governs reproduction in TCM theory, and patients or experimental animals with KYDS exhibit imbalance in hypothalamus-pituitary- gonadal axis (HPG), which is closely associated with reproduction. E2 and T, key markers for HPG, are used to evaluate the KYDS model [30–33]. Antagonism between cAMP and cGMP is similar to the opposition of yin and yang in TCM theory, and these two markers are commonly used to assess the deficiency syndrome [21, 24, 31, 34]. Therefore, the body weight, temperature, and serum levels of T_3_, T_4_, E_2_, T, cAMP, and cGMP were selected as the indices for evaluating the severity of KYDS. In the present study, the rats intragastrically administered with L-T_4_ in the KYDS group exhibited dysphoria. At the same time, the treated rats showed reduced body weight and higher body temperature relative to the rats in the control group. Furthermore, rats in the KYDS group showed significantly higher levels of T_3_, T_4_, cAMP, cAMP/cGMP, and E_2_, but markedly lower T levels relative to the control group. These clinical manifestations were similar to those reported in the KYDS rats in previous studies, which indicated that the KYDS rat model was successfully duplicated.

To elucidate the pathological essence and mechanisms underlying L-T_4_ induced KYDS, we identified the DEPs in the in hypothalamus by iTRAQ-based proteomics. Results of GO analysis revealed that the majority of the DEPs were primarily involved in single-organism metabolism process in BP, extracellular region and organelle in CC, and oxidoreductase activity in MF. Biological pathway analysis could also reveal important insights into KYDS. Fatty acid degradation (rno00071) and pyruvate metabolism (rno00620) were found to be enriched in KYDS. Although fatty acids are not the primary energy source for the utilization of the brain, the intermediates of fatty acid metabolism serve as hypothalamic sensors for energy status [35]. Pyruvate, which realizes mutual transformation of glucose, fat, and amino acid through acetyl-CoA and tricarboxylic acid cycle, is a pivotal intermediate in carbohydrate metabolism and acts as an important hub in the metabolic pathway [36].

In TCM clinical practice, the typical manifestations of KYDS patients include glucose metabolism disorder, which is closely related to fatty acid metabolism in the hypothalamus [2, 37]. The hypothalamus acts as a metabolic regulatory center and exerts distinct effects on glucose homeostasis and energy metabolism [38]. Our results indicated that the expression levels of Acaa2, Acsl1, and Hadha, which are closely related to fatty acid degradation, were altered in hypothalami of KYDS rats. The hypothalamus is known to detect the presence of long-chain fatty acids as a signal for nutrition surplus, and hypothalamic fatty acid metabolism is involved in regulating overall lipid and glucose balance in the body [39, 40]. Long-chain acyl-CoA synthetases (ACSLs) catalyze the first step of fatty acid metabolism by converting long-chain fatty acid (LCFA) into acyl-CoA thioesters [41]. Acsl1, which belongs to the family of ACSLs, plays a crucial role in lipid biosynthesis and fatty acid metabolism [42]. Acsl1 is located on the outer mitochondrial membrane and converts LCFA into long-chain fatty acid-CoA (LCFA-CoA) through esterification [43]. The downregulation of Acsl1 levels in the hypothalamus inhibits the esterification of LCFA, thereby reducing the generation of LCFA-CoA [44]. The accumulation of LCFA-CoA in the hypothalamus has been verified to decrease hepatic glycogen infusion [45, 46]. Thus, low levels of LCFA-CoA in hypothalamus will promote hepatic glucose production. Trifunctional enzyme is located in the inner mitochondrial membrane and catalyzes three consecutive steps in the mitochondrial long-chain fatty acid *β*-oxidation process [47]. Trifunctional enzyme possesses two subunits, namely, the alpha subunit (Hadha), which catalyzes the hydration of 3-hydroxyacyl-CoA dehydrogenase and enoyl-CoA hydratase activities, with beta subunit (Hadhb) catalyzing the 3-ketoacyl-CoA thiolase activity [48]. Acaa2, also called acetyl-CoA acyltransferase 2, is a mitochondrial enzyme that catalyzes the last step of fatty acid oxidation to produce acetyl-CoA needed for the citrate cycle [49]. In this study, the expression levels of Acsl1, Hadha, and Acaa2 in the hypothalamus were found to be significantly downregulated in the KYDS group relative to those in the control group. These three proteins are known to play significant roles in fatty acid *β*-oxidation. In turn, the downregulation of the three proteins inhibit fatty acid *β*-oxidation and dampen fatty acid degradation. The above findings indicated that abnormal glucose metabolism observed in KYDS rats were caused by reduced LCFA-CoA levels and fatty acid degradation mediated by the downregulation of Acsl1, Hadha, and Acaa2 levels.

Thyroxine significantly influences glucose metabolism in the body [50]. Abnormal blood glucose levels always occur in KYDS patients and the L-T_4_-induced KYDS rats [5, 51, 52]. Most notably, the current findings revealed that Ldha and Pck1 are involved in pyruvate metabolism. Pyruvate metabolism is a key step in glycolysis or gluconeogenesis, moreover, glucose is the main energy source in the brain [53]. Pck1 is a rate-limiting enzyme that catalyzes the conversion of oxaloacetate to phosphoenolpyruvate, which subsequently converted into pyruvate by pyruvate dehydrogenase [54]. Herein, the expressions of Pck1 were found to decrease in the KYDS rats. The reduced Pck1 will indirectly suppress the production of pyruvate. Ldha belongs to the family of lactate dehydrogenase and is known to catalyze the conversion of pyruvate into lactate during glycolysis [55]. Lactate is a key intermediate and exerts distinct biological effects in the brain. Lactate is the main energy source in the brain when abnormal glucose metabolism of the brain occurs. The activation of lactate metabolism in hypothalamus leads to lower the glucose levels in the liver and plasma [56]. The expression of Ldha was found to decline in the KYDS group compared to the control group, and the reduction in Ldha levels could inhibit the formation of lactate from pyruvate, thereby impairing lactate metabolism. In turn, dysregulated lactate metabolism in the hypothalamus increases hepatic glucose levels, which accounts for the glucose metabolic disorders during KYDS.

## 5. Conclusions

In the present study, we identified a large number of differently expressed proteins in the hypothalami of L-T_4_ induced KYDS rats based on iTRAQ-based 2D-LC-MS/MS. In addition, RT-PCR analysis validated the different expressions of Acsl1, Hadha, Acaa2, Pck1, and Ldha at mRNA level. Taken together, the current findings indicated that metabolic disorders related to glucose and lipid are associated with the inhibition of fatty acid degradation and pyruvate metabolism. We speculated that Acsl1, Hadha, Acaa2, Pck1, and Ldha in the hypothalamus play critical roles in mediating lipid and glucose metabolic disturbances in the development of KYDS, and these proteins serve as potential biomarkers for KYDS. This present study identified novel biomarkers in KYDS rats, which provided some new clues for further research on the pathological essence of KYDS in TCM.

## Figures and Tables

**Table 1:** Gene specific primers used for RT-PCR.

Gene name	Specific primers
GAPDH-F	GGGTGTAACCACGAGAAAT
GAPDH-R	ACTGTGGTCATGAGCCCTTC
ACSL-F	GGTGCTTCAGCCTACCATCTTCC
ACSL-R	AATCCAACAGCCATCGCTTCACT
ACAA2-F	AAGCTGATCCCACTGCGTATTT
ACAA2-R	ACGTGAGTGGAGGTGCCATAG
HADHA-F	TCAAGGACGGACCTGGCTTCTAC
HADHA-R	TTCTGCTACGTGCTGTGCTACATC
PCK1-F	TGTTGGCTGGCTCTCACTG
PCK1-R	ACTTTTGGGGATGGGCAC
LDHA-F	CCGTTACCTGATGGGAGAAA
LDHA-R	ACGTTCACACCACTCCACAC

## Data Availability

All datasets analyzed during the current study are available from the corresponding author upon reasonable request.

## Abbreviations

KYDS: Kidney-yin deficiency syndrome
TCM: Traditional Chinese Medicine
L-T_4_: Levothyroxine
T_3_: Triiodothyronine
T4: Thyroxine
iTRAQ: Isobaric tags for relative and absolute quantitation
DEPs: Different expressed proteins
GO: Gene Ontology
BP: Biological process
Estrogen: E_2_
CC: Cellular component
MF: Molecular function
KEGG: Kyoto Encyclopedia of Gene and Genomes
Acaa2: 3-ketoacyl-CoA thiolase
Acsl1: Long-chain acyl-CoA synthetase 1
Hadha: Trifunctional enzyme subunit alpha
Pck1: Cytosolic phosphoenolpyruvate carboxykinase
Ldha: L-lactate dehydrogenase A chain
ELISA: Enzyme-linked immunosorbent assay
Testosterone: T.

## Supplementary Materials

Supplementary Materials The workflow of this research has been embedded in the supplementary materials; “see Figure S1 in supplementary materials”.
