# Supplementary material for: iTRAQ-Based Proteomics to Reveal the Mechanism of Hypothalamus in Kidney-Yin Deficiency Rats Induced by Levothyroxine
Source: Evid Based Complement Alternat Med. 2019 Mar 4;2019:3703596. doi: 10.1155/2019/3703596 (PMC6425355; doi:10.1155/2019/3703596)
Supplement: Supplementary Materials — The workflow of this research has been embedded in the supplementary materials; “see Figure S1 in supplementary materials”. [file 3703596.f1.docx]

**Supporting information**

**Evidence-Based Complementary and Alternative Medicine**

**iTRAQ-based proteomics to reveal the mechanism of hypothalamus in Kidney yin deficiency rats induced by levothyroxine**

Wei Guan,^1,ϯ^ Yan Liu,^1,ϯ^ Xiaomao Li,^1^ Bingyou Yang,^1,*^ and Haixue Kuang ^1,*^

^1^ Key Laboratory of Chinese Materia Medica (Ministry of Education), Heilongjiang University of Chinese Medicine, Harbin 150040, China

Correspondence should be addressed to Bingyou Yang; ybywater@163.com, Haixue Kuang; [hxkuang@yahoo.com](mailto:hxkuang@yahoo.com)

ϯWei Guan and ϯYan Liu contributed equally to this work.


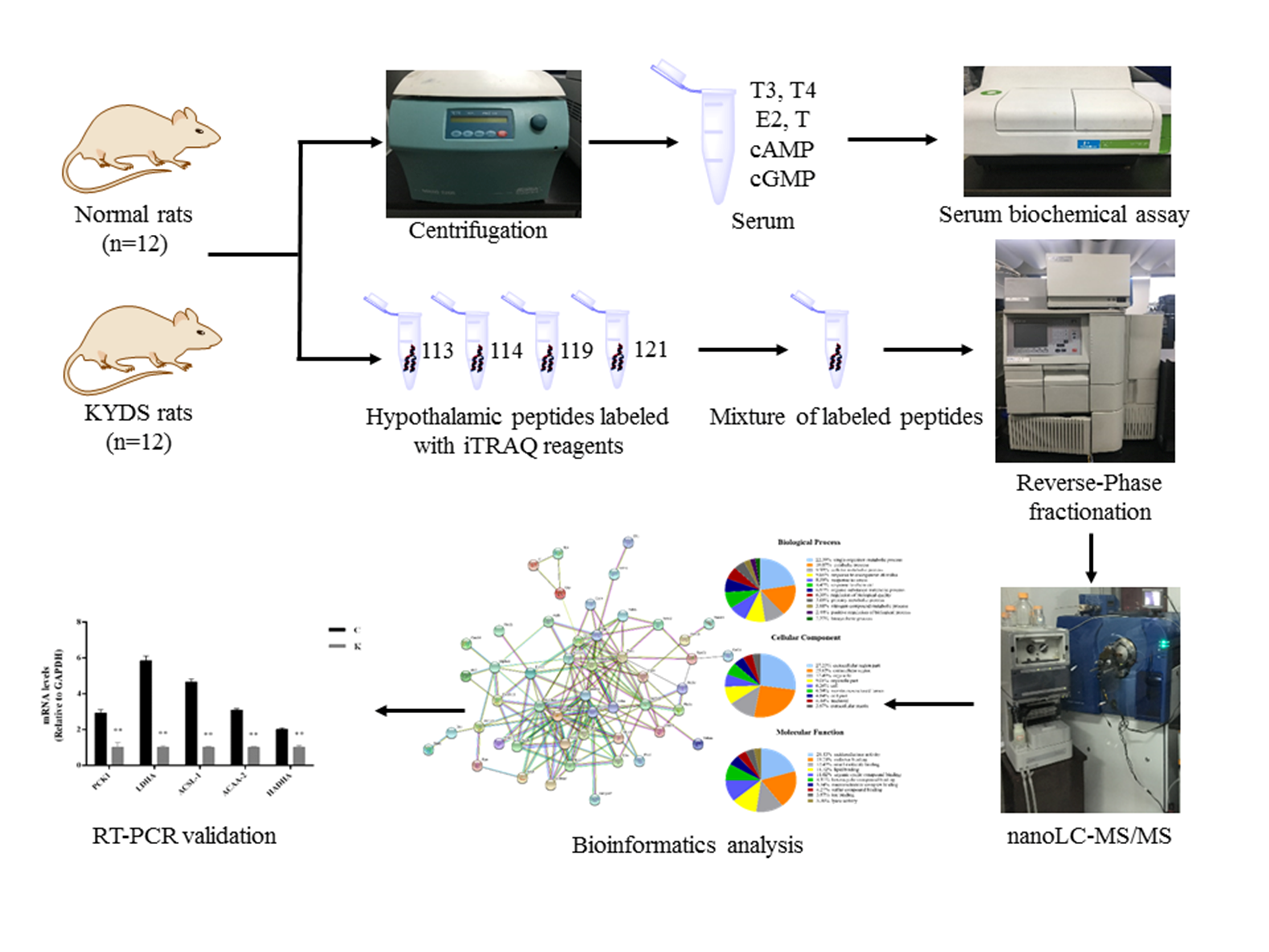


Figure S1. The workflow of the investigation of hypothalamic mechanism in KYDS rats by iTRAQ-based proteomics.
